# The European registry of quality outcomes for cataract and refractive surgery (EUREQUO): a database study of trends in volumes, surgical techniques and outcomes of refractive surgery

**DOI:** 10.1186/s40662-015-0019-1

**Published:** 2015-04-30

**Authors:** Mats Lundström, Sonia Manning, Peter Barry, Ulf Stenevi, Ype Henry, Paul Rosen

**Affiliations:** Department of Clinical Sciences, Ophthalmology, Faculty of Medicine, Lund University, Lund, Sweden; Department of Ophthalmology, Mater Misericordiae University Hospital, Dublin, Ireland; Department of Ophthalmology, St. Vincent’s University Hospital, Dublin, Ireland; Department of Ophthalmology, Sahlgren’s University Hospital, Molndal, Sweden; Department of Ophthalmology, Vumc, Amsterdam, The Netherlands; Oxford Eye Hospital, Oxford, UK

**Keywords:** Refractive surgery, Registry, European database, Outcomes

## Abstract

**Background:**

A European web-based registry for refractive surgery was established in 2008; The European Registry of Quality Outcomes for Cataract and Refractive Surgery (EUREQUO). The aim of the registry was to improve treatment and standards of care for refractive surgery. Further aims were to offer a tool for benchmarking by establishing a reference database and for surgeons to enter and analyze their own outcomes. The purpose of this study was to characterize the registry and analyze the data collected during its first decade.

**Methods:**

The characteristics of the web-based registry are described. Data collected from February 4^th^ 2004 until June 30^th^ 2014 are included in the analysis. The database is analyzed in terms of surgical technique, indications for surgery, complications, and refractive and visual outcomes.

**Results:**

Data have been reported from 47 centers in 14 countries until mid-2014. About 4,000 procedures were reported annually. The most frequent procedure was laser-assisted in-situ keratomileusis (LASIK) with 11697 reported surgeries. Over time in the database, LASIK declined (p < 0.001) while photorefractive keratectomy (PRK) and refractive lens exchange (RLE) increased (p < 0.001 for both procedures). The indications for surgery, in terms of preoperative refraction and age, were stable over time, for all types of procedures. Surgical complications were reported infrequently and with a well-known relationship to the type of surgical procedure. The reported refractive outcomes were good. The visual outcomes indicate a significant increase of visual acuity after high myopia treatment by phakic intraocular lens in the anterior (phakic IOL AC) and the posterior (phakic IOL PC) chamber and a poorer visual outcome, after both myopia and hyperopia treatment, by epithelial LASIK (Epi-LASIK).

**Conclusions:**

We describe the establishment of a European registry for refractive surgery. The database increases at a rate of approximately 4000 refractive procedures per year. The most frequent procedure is LASIK, but both PRK and RLE are an increasing part of the reported procedures. The indications for surgery have been stable over time. Surgical complications and visual outcome vary, depending on the type of surgery.

## Background

In 2004, the European Society of Cataract & Refractive Surgeons (ESCRS) took the initiative to establish a registry for refractive surgery outcomes: the Refractive Surgery Outcomes Information System (RSOIS). The purpose of this web-based system was to record outcomes of refractive surgery and improve quality of care for these procedures. Reasons behind the initiative were the growing health tourism within the field and increasing patient complaints after refractive surgery reported in the press, in some countries [1,2]. Patient complaints were thought to be associated with inappropriate indications and surgery outside the limits of the procedure, leading to suboptimal outcomes in refractive surgery. In addition, it is well known that monitoring outcomes, in cataract surgery, can make surgeons more aware of quality control, which can lead to improved outcomes [3]. A refractive surgery outcomes registry might reasonably be expected to have the same influence on outcomes in refractive surgery. The number of refractive surgeries entered into the system increased slowly during the following years (personal communication P. Barry). In 2007, the ESCRS applied for an EU grant to create a European registry, aimed at improving the quality of both cataract and refractive surgery. The philosophy behind the project was to create a database for learning and quality improvement, not for supervision. The EU grant application was successful, and led to the creation of the European Registry of Quality Outcomes for Cataract and Refractive Surgery (EUREQUO) [4,5] with two co-financers: the European Union under the Executive Agency for Health and Consumers, and the ESCRS. The ESCRS was the lead partner in the project, and eleven national societies participated as associated partners.

The aims of the project were to improve treatment and standards of care for cataract and refractive surgery, and to develop evidence-based guidelines for cataract and refractive surgery across Europe. Further aims were to offer a tool for benchmarking by establishing a reference database and to provide a system for surgeons to record and analyze their own outcomes. Finally, the project also served to continue the RSOIS initiative and merge data into the new database. Details about technical, clinical, and legal issues have been published earlier [4].

The purpose of this report is to describe the establishment of a European refractive surgery database and to describe the achievements, in terms of participating countries, surgeons, and trends within refractive surgery, as reflected by the growing database.

## Methods

**Figure 1:**
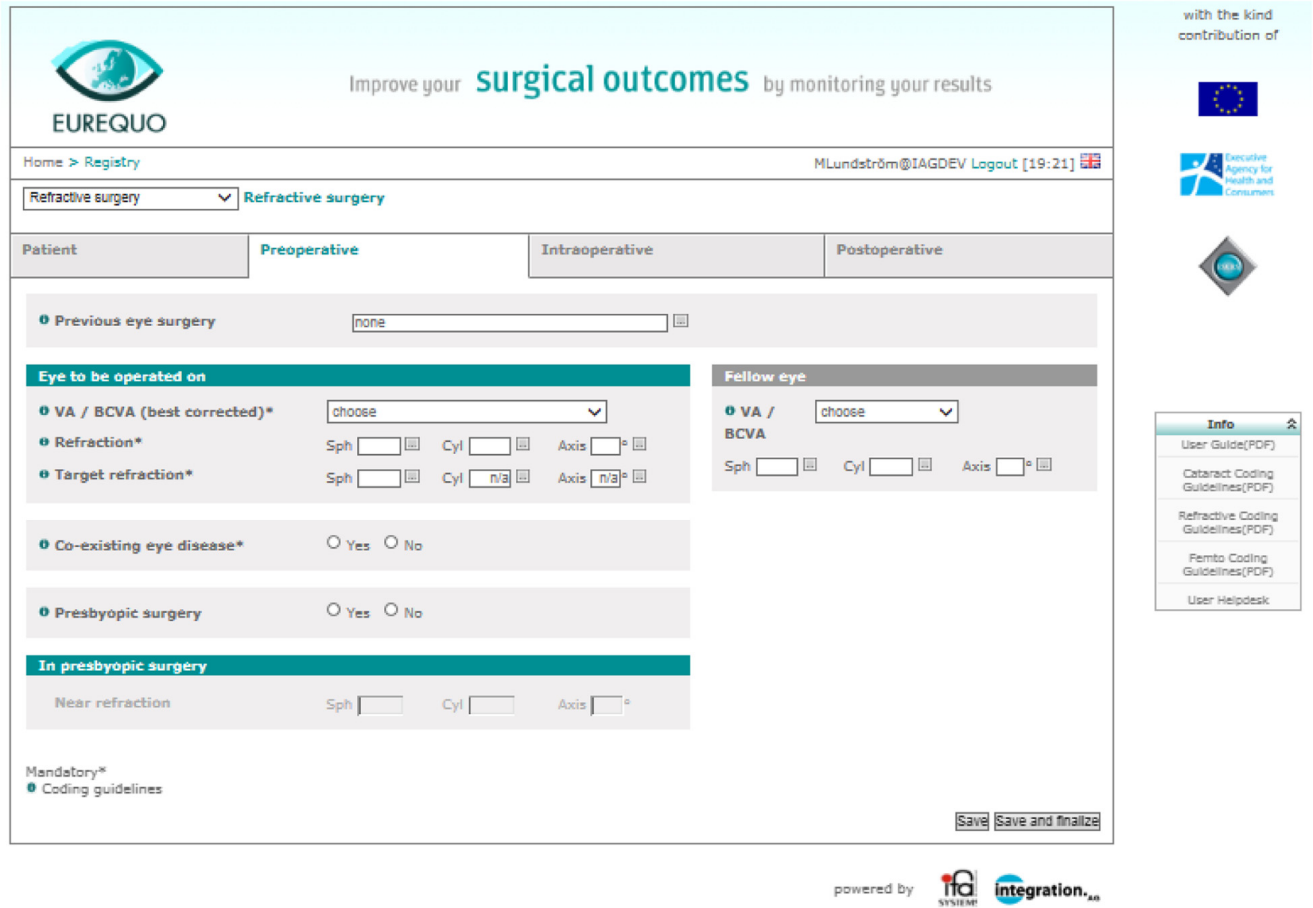
Web form for entering surgical data. This form is for preoperative data.

A web-based system was created for input and output of data. The system can be accessed via the EUREQUO web portal (http://eurequo.org) for manual input of data (Figure 1). Individual patient data are anonymous in the registry, but for auditing purposes each participating center guarantees traceability of a registered surgical procedure back to the corresponding medical record in the center. Data can also be entered into the database from existing national registries or electronic medical record (EMR) systems. The web-based system has built-in security features for data protection. No field allows the entry of free text or numbers. Instead, possible values/options for a certain parameter are selected from a matrix/drop-down menu, on the web page. The database also contains built-in data error checks, which will not allow impossible values for biometric parameters or dates in incorrect chronological order (e.g. follow-up date before surgery date, etc.) to be entered.

**Figure 2:**
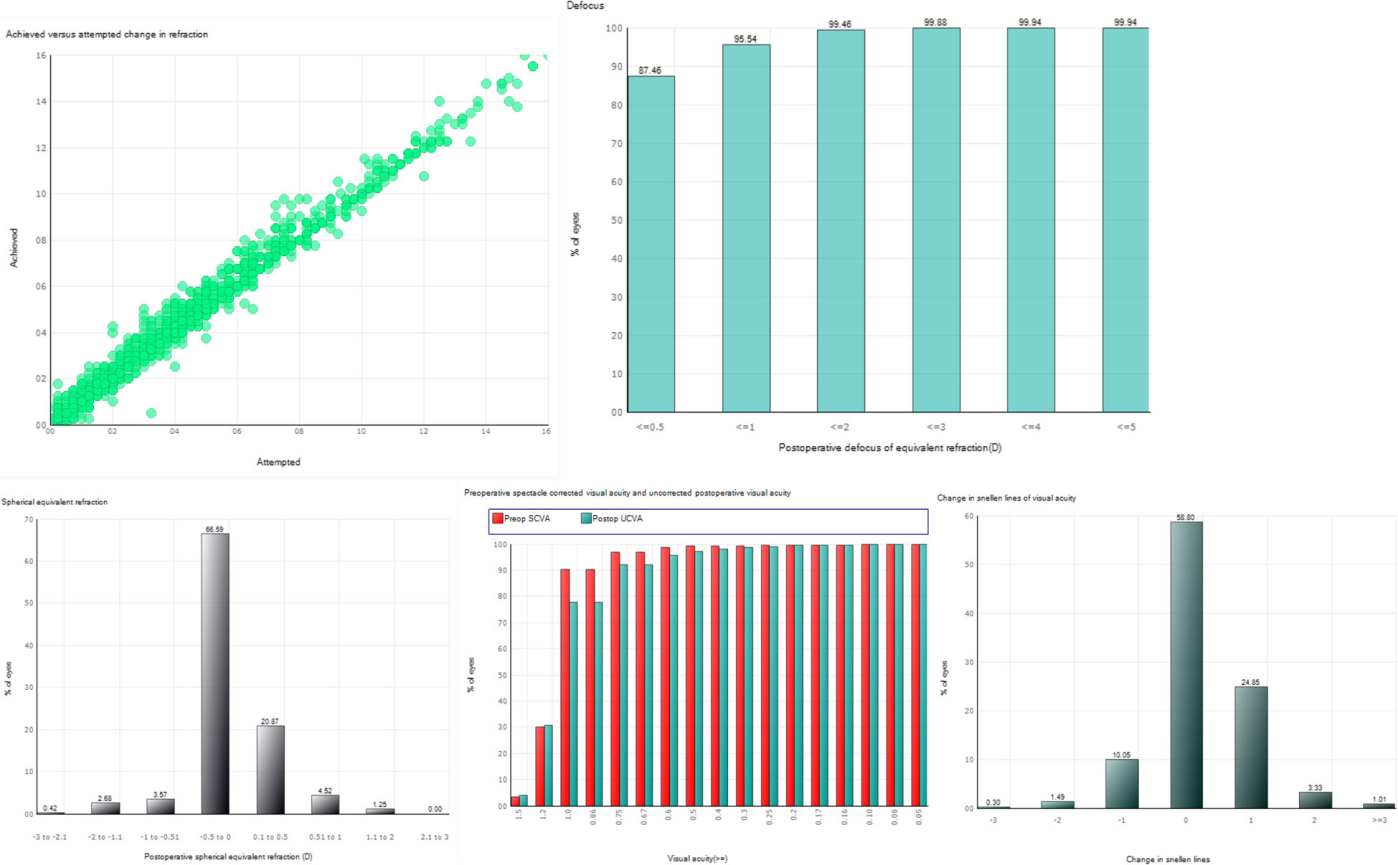
Standard graphs showing the surgical outcomes for one clinic or surgeon. The upper left graph shows attempted refraction versus achieved refraction in Diopters. The upper right graph shows postoperative defocus of equivalent refraction in Diopters. The lower left graph shows the postoperative spherical equivalent refraction. The lower mid graph shows the preoperative spectacle corrected visual acuity versus the postoperative uncorrected visual acuity. Both bar rows show the accumulated frequency of visual acuity. The lower right graph shows the visual outcome in terms of loss of one Snellen line or more, status quo or gain of one Snellen line or more.

In refractive surgery, the goal is to achieve optimal visual acuity, optimal refraction (usually emmetropia), and no complications. Quality indicators reflecting these three important outcomes were decided on, and dictated the choice of variables. Demographic and case-mix variables were also included for accurate benchmarking. The output reports were designed on the basis of earlier experience (RSOIS). Each participating center/surgeon can obtain output statistics from the database via the web, in the form of frequency tables or graphs (Figure 2). The system offers one report including preoperative and intraoperative data and one report including follow-up data. It is also possible to export one’s own data from the system as an Excel file. Each center/surgeon can only obtain access to their own data, aggregated data for any participating country, and aggregated data for the whole database. This means that the output data for a particular center/surgeon can only be accessed by that center/surgeon and also forms part of the aggregated data for their country and for the whole database, which is accessible to all participating centers/surgeons. The output tables and graphs are standardized with the option of using the preoperative variables of gender, age, date of surgery, type of operation, co-morbidity, and complex surgery, as filters, allowing the participating center/surgeon to isolate and analyze specific cohorts among their reported cases. While this covers most of the purposes for which data output is required, export of data as an Excel file is also possible, allowing the center/surgeon to make their own customized output reports and perform advanced statistical procedures. Follow-up data must be reported within 6 months. There are coding guidelines for how to report data on the web-forms [6].

The data presented here have been derived from the EUREQUO database. Data have been entered into the system from February 4^th^ 2004 (in the RSOIS database during 2004-2007 and in the EUREQUO database from 2008 onwards). Data entered until June 30^th^ 2014 are included in the analysis. All statistical calculations were performed using IBM SPSS Statistics software package, version 22, IBM Ltd, Chicago, Ill. Changes over time for surgical techniques were tested with logistic regression analysis. A p-value of <0.05 was considered significant.

## Results

The number of refractive procedures reported into the system between February 4^th^, 2004 and June 30^th^, 2014 was 27,339. Data were entered by 47 centers/surgeons from 14 countries. The contributing countries are Denmark, Austria, Germany, Greece, Great Britain, Ireland, Spain, Iceland, Belgium, The Netherlands, Slovakia, Slovenia, Sweden and Turkey.

The number of refractive procedures reported to the database, per annum, from 2010 onwards, was, approximately, 4000.

The most frequently reported procedure in the database was Laser-assisted in-situ keratomileusis (LASIK) (Table 1).

**Table 1:** **Number of refractive procedures (eyes), reported into the database, between February 4** ^**th**^ **2004 and June 30** ^**th**^ **2014**

**Refractive procedure**	**Number of procedures**	**Procedures in per cent**
LASIK	11697	44.0
LASEK	5342	20.1
PRK	4656	17.5
Phakic IOL AC	1376	5.2
RLE	1155	4.3
Epi-LASIK	877	3.3
Phakic IOL PC	316	1.2
Other	1179	4.4

LASIK = Laser-assisted in-situ keratomileusis, LASEK = Laser-Assisted Subepithelial Keratectomy, PRK = Photorefractive Keratectomy, Phakic IOL AC = Phakic intra-ocular lens in anterior chamber, RLE = Refractive Lens Exchange, Epi-LASIK = Epithelial LASIK, Phakic IOL PC = Phakic intra-ocular lens in posterior chamber.

**Figure 3:**
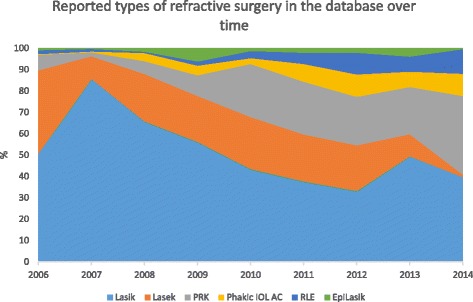
Surface diagram showing database trend of reported type of surgical procedure. The diagram does not include the first two years because of the limited number of cases and clinics.

The majority (95.2%) of reported refractive procedures were primary procedures. The remaining procedures were enhancements or additional procedures to a primary procedure. The frequency of reported surgical techniques, changed over time (Figure 3). The proportion of LASIK procedures decreased (p < 0.001), while photorefractive keratectomy (PRK), refractive lens exchange (RLE) and anterior chamber intra-ocular lenses (Phakic IOL AC) increased (p < 0.001), over the study period.

The indication for surgery in terms of preoperative spherical refraction and age, differed among surgical techniques and between myopia and hyperopia. Table 2 shows the indication, mean age and preoperative refraction for the most frequently reported refractive procedures in the database.

**Table 2:** **Type of (primary) procedure, indication, mean age and range of preoperative refraction**

**Type of procedure**	**Indication**	**Mean age at surgery (years)**	**Maximum myopic refraction**	**Mean myopic refraction**	**N**
LASIK	Myopia	35	−12.0	−3.55	8405
LASEK	Myopia	34	−11.75	−3.38	4738
PRK	Myopia	34.2	−9.5	−3.2	3693
Phakic IOL AC	Myopia	38	−20.0	−8.5	1010
Epi-LASIK	Myopia	35.6	−9.5	−3.35	418
RLE	Myopia	54.2	−20.0	−5.8	323
			**Maximum hyperopic refraction**	**Mean hyperopic refraction**	
LASIK	Hyperopia	44	+15.00	2.40	1688
RLE	Hyperopia	56.5	+13.0	3.4	792
PRK	Hyperopia	44.6	+7.25	1.5	308
LASEK	Hyperopia	43	+6.50	1.93	273
Phakic IOL AC	Hyperopia	33.9	+10.50	5.1	124
Epi-LASIK	Hyperopia	38.5	+6.50	1.90	35

LASIK = Laser-assisted in-situ keratomileusis, LASEK = Laser-Assisted Subepithelial Keratectomy, PRK = Photorefractive Keratectomy, Phakic IOL AC = Phakic intra-ocular lens in anterior chamber, Epi-LASIK = Epithelial LASIK, RLE = Refractive Lens Exchange, N = number of procedures.

Refraction in Diopters.

In the refractive correction of myopia, LASIK was mainly used for low myopic corrections, with 86% of the treated eyes in the database having preoperative spherical equivalent of less than -6.0 D. For laser-assisted subepithelial keratectomy (LASEK) and PRK, the corresponding numbers were 87.6% and 89.7%, respectively. Phakic IOL AC were used for higher myopic corrections; 31.3% of reported phakic IOL AC cases had preoperative spherical equivalent of -10.0 D or more, and 83.3% had -6.0 D or more. The corresponding numbers for RLE were 13% and 47.1%, respectively.

Trends over time, in the level of preoperative myopia, as an indication for specific refractive procedures, are shown in Table 3.

**Table 3:** **Mean and maximum preoperative refraction, of myopic and hyperopic patients, who underwent various refractive procedures**

**Type of refractive procedure**	**Preoperative myopic refraction (D), reported per year**									
		**2006**	**2007**	**2008**	**2009**	**2010**	**2011**	**2012**	**2013**	**2014**
LASIK	Mean	−3.9	−3.2	−3.5	−3.3	−3.5	−3.7	−3.5	−3.4	−3.2
	Maximum	−11.25	−12.0	−10.0	−11.0	−10.0	−10.25	−11.75	−10.25	−8.25
PRK	Mean	−3.9	−2.3	−3.7	−3.1	−2.9	−3.1	−3.5	−3.2	−3.3
	Maximum	−9.5	−5.5	−4.25	−8.0	−9.0	−9.5	−9.25	−8.5	−8.5
RLE	Mean	−6.7	*	*	−6.2	−5.9	−5.9	−4.7	−6.0	−7.1
	Maximum	−14.0	*	*	−6.5	−10.0	−11.75	−19.0	−20.0	−15.5
Phakic IOL AC	Mean	*	*	−7.4	−8.6	−10.3	−8.4	−8.5	−9.0	−8.3
	Maximum	*	*	−10.25	−15.25	−16.5	−20.0	−20.0	−20.0	−15.75
Epi-LASIK	Mean	−4.4	*	*	−3.5	−3.3	−2.8	−3.5	−3.25	*
	Maximum	−8.25	*	*	−6.25	−6.25	−8.0	−8.25	−9.5	*
LASEK	Mean	−3.2	−2.5	−4.4	−4.0	−3.4	−3.5	−3.7	−3.5	*
	Maximum	−10.5	−7.25	−10.5	−9.0	−10.0	−11.5	−11.25	−11.75	*
**Type of refractive procedure**	**Preoperative hyperopic refraction (D), reported per year**									
		**2006**	**2007**	**2008**	**2009**	**2010**	**2011**	**2012**	**2013**	**2014**
LASIK	Mean	2.7	2.1	2.5	2.4	2.5	2.4	2.2	2.3	2.5
	Maximum	15.0	5.8	5.8	6.5	6.3	7.3	6.0	6.5	5.5
PRK	Mean	*	*	*	*	1.4	1.4	1.5	1.2	*
	Maximum	*	*	*	*	4.3	3.2	4.5	7.3	*
RLE	Mean	4.4	*	*	*	3.1	3.4	3.0	3.2	4.5
	Maximum	9.5	*	*	*	9.5	9.8	10.8	10.0	10.3
Phakic IOL AC	Mean	*	*	*	*	*	5.2	4.9	5.2	*
	Maximum	*	*	*	*	*	10.3	8.3	10.5	*
LASEK	Mean	2.1	*	*	*	1.8	1.6	2.0	*	*
	Maximum	6.0	*	*	*	4.5	6.0	6.5	*	*

* = Too few cases for statistical analysis, data given only if N > 30.

LASIK = Laser-assisted in-situ keratomileusis, PRK = Photorefractive Keratectomy, RLE = Refractive Lens Exchange, Phakic IOL AC = Phakic intra-ocular lens in anterior chamber, Epi-LASIK = Epithelial LASIK, LASEK = Laser-Assisted Subepithelial Keratectomy.

The table shows trend, over time, in the database.

The mean preoperative refraction for different reported refractive procedures, was stable over time, in the database (Table 4).

**Table 4:** **Mean age of patients with myopia who underwent a refractive procedure**

**Type of refractive procedure**	**Mean age of patients (yrs), reported per year**								
	**2006**	**2007**	**2008**	**2009**	**2010**	**2011**	**2012**	**2013**	**2014**
LASIK	36.5	30.5	32.4	36.1	34.6	36.4	35.1	35.7	37.3
PRK	33.0	33.1		34.4	34.8	35.4	34.2	33.2	32.7
RLE	51.7	*	*	51.4	52.6	52.6	56.2	53.8	56.6
Phakic IOL AC	*	*	36.8	36.2	37.8	38.7	38.2	36.6	37.2
LASEK	33.7	35.1	38.4	37.8	35.2	34.5	33.0	36.2	*
Epi-Lasik	34.4	*	*	35.6	36.8	36.2	33.6	33.6	*

* = Too few cases for statistical analysis, data given only if N > 30.

LASIK = Laser-assisted in-situ keratomileusis, PRK = Photorefractive Keratectomy, RLE = Refractive Lens Exchange, Phakic IOL AC = Phakic intra-ocular lens in anterior chamber, LASEK = Laser-Assisted Subepithelial Keratectomy, Epi-LASIK = Epithelial LASIK.

The table shows trend over time, in the database.

The mean age of patients undergoing RLE increased (p < 0.001, linear regression) over time, but the mean age of patients undergoing other refractive procedures, remained stable.

Surgical and postoperative complications are listed in Table 5.

**Table 5:** **Surgical and postoperative complications (%)**

	**LASIK**	**LASEK**	**PRK**	**RLE**	**Phakic IOL AC**	**Epi-LASIK**
**Intraoperative complications (%)**	**0.5**	**0.6**	**0.3**	**1.1**	**0.6**	**0.4**
***Corneal complications***	**0.5**	**0.6**	**0.3**	**0.2**	**0.1**	**0.4**
Flap-related	0.4	0.2	0	0	0	0
Implant-related	0	0	0	0	0	0
Other	0.1	0.4	0.3	0.2	0	0.4
***Intraocular complications***	**0**	**0**	**0**	**0.9**	**0.5**	**0**
PCR	0	0	0	0.3	0	0
Endothelium	0	0	0	0	0	0
Iris	0	0	0	0.2	0.3	0.
Other	0	0	0	0.4	0.3	0
**Postoperative complications (%)**	**1.2**	**2.7**	**1.6**	**2.6**	**4.8**	**4.2**
DLK	0.3	0	0	0	0	0
Implant-related	0	0	0	0.2	0.7	0
Corneal edema	0	0	0	0.1	0.1	0
Optical error	0.4	0.9	0	1.1	0.3	0
Haze	0	1.5	1.2	0	0	3.6
Elevated IOP	0	0.1	0	0.1	2.1	0
Infection	0	0	0	0	0.1	0
Retinal	0	0	0	0.3	0.5	0
Ectasia	0	0	0	0	0	0
Cataract	0	0	0	0.1	0.1	0
Other	0.5	0.2	0.3	0.9	1.2	0.7
**All complications (%)**	**1.7**	**3.3**	**1.9**	**3.7**	**5.4**	**4.6**

LASIK = Laser-assisted in-situ keratomileusis, LASEK = Laser-Assisted Subepithelial Keratectomy, PRK = Photorefractive Keratectomy, RLE = Refractive Lens Exchange, Phakic IOL AC = Phakic intra-ocular lens in anterior chamber, Epi-LASIK = Epithelial LASIK, PCR = Posterior Capsule Rupture, DLK = Diffuse Lamellar Keratitis, IOP = intraocular pressure.

Table 5 shows that Epi-LASIK had the highest reported rate of postoperative haze, while Phakic IOL AC had the highest reported rate of elevated intraocular pressure (IOP), following surgery. Refractive lens exchange had the highest reported rate of intraoperative complications, while LASIK and PRK had the lowest reported rate of all complications.

The mean postoperative refraction (spherical equivalent), achieved with different refractive procedures, is shown in Table 6. The table only includes cases in which the target postoperative refraction was 0 Diopters (D).

**Table 6:** **Mean refractive outcome of patients undergoing various refractive procedures**

**Refractive procedure**	**Myopic treatment**		**Hyperopic treatment**	
	**Mean postoperative refraction**	**STD**	**Mean postoperative refraction**	**STD**
LASIK	0.041	0.517	0.172	0.638
LASEK	0.057	0.409	0.028	0.593
PRK	0.006	0.397	−0.053	0.777
Epi-LASIK	−0.032	0.308	0.000	0.177
RLE	0.051	0.460	0.185	0.513
Phakic IOL AC	0.015	0.735	−0.036	0.470

STD = Standard Deviation, LASIK = Laser-assisted in-situ keratomileusis, LASEK = Laser-Assisted Subepithelial Keratectomy, PRK = Photorefractive Keratectomy, Epi-LASIK = Epithelial LASIK, RLE = Refractive Lens Exchange, Phakic IOL AC = Phakic intra-ocular lens in anterior chamber.

Only the cases in which the target postoperative refraction was emmetropia, were included. Follow-up was 1-6 months. Refraction in Diopters.

As shown in Table 6, the mean refractive outcome within a follow-up time of 1 to 6 months is reported as very good for all surgical techniques. Table 7 shows the change in visual acuity for myopic and hyperopic treatments, for various refractive procedures.

**Table 7:** **Change (%) in visual outcome for myopic and hyperopic treatments, for various refractive procedures (eyes)**

**Refractive procedure**	**Myopic treatment**			**Hyperopic treatment**		
**Change in visual acuity**	**Loss of 1 or more lines**	**Unchanged**	**Gain of 1 or more lines**	**Loss of 1 or more lines**	**Unchanged**	**Gain of 1 or more lines**
LASIK	15.1	62.7	22.1	20.3	62.2	17.5
LASEK	14.7	66.7	18.5	13.1	72.4	14.6
PRK	11.8	65.7	22.6	13.0	65.1	21.8
Epi-LASIK	28.1	57.6	14.3	37.1	60.0	14.3
RLE	8.0	52.3	39.6	18.7	55.7	25.6
Phakic IOL AC	7.2	50.6	42.2	16.9	59.7	23.4

Table 7 shows a good reported visual outcome for treating high myopia with Phakic IOL AC or RLE. Poorest visual outcome is reported for treating hyperopia with Epi-LASIK.

## Discussion

In this study, LASIK is the most frequently reported refractive surgery. LASIK technique and outcome has indeed been reported in numerous studies since the development of this surgical technique [7,8]. However, data from real world practice (i.e., from registries) in different countries have been scarce. Therefore, no one has determined the number of LASIK procedures that has been carried out in various countries. In the EUREQUO database, the frequency of a certain surgical technique may, of course, depend on which clinics and countries data is reported from. The second most frequently reported surgical procedure is LASEK. However, the trend in the database is a strong decrease of LASEK procedures over time. One reason for this may be fear for haze as a postoperative complication [9]. The third most commonly reported procedure is PRK. PRK takes an increasing part of the reported procedures in the database. The distinction between LASEK and PRK has become blurred: they are, essentially, the same procedure, but in the former, the epithelium is retained, while in the latter, it is removed. It is possible that the changing use of each is because surgeons no longer felt there was a benefit to LASEK and that LASEK procedures were converted preoperatively into PRK. The additional effect of the changing trends for these three procedures probably reflects the fact that they all are used for the same indication: low and medium myopia. An evident change over time is the increasing number of RLE cases. This procedure offers some advantages compared with corneal refractive surgery. It is indicated in older presbyopic patients, hyperopes, and those with early cataract formation. The age group undergoing a refractive procedure is older, due, in part, to the ability to correct presbyopia, but they are also economically better-off, and can afford the treatment [10].

The majority of refractive procedures in the database were aimed at treating myopia. Low and medium myopia was reported for LASIK, LASEK, Epi-LASIK and PRK. These techniques were used for eyes with an average myopia between -3.2 to -3.6 D. For higher myopia, Phakic IOL (average -8.5 D) or RLE (average -5.8 D) were used. Trends in the database do not show significant changes in indications for treating myopia or hyperopia over time (Table 3).

Complications during and after surgery are of distinct concern as the eyes undergoing refractive surgery are usually healthy eyes. PRK is more related to corneal complications compared with LASIK [11]. In this study, corneal complications in PRK are not reported in a higher degree than LASIK except haze that is reported in 1.2% versus 0%, respectively. LASEK and Epi-LASIK was reported to have a higher occurrence of postoperative haze in this study: 1.5% and 3.6%, respectively. Higher frequency of haze after these two types of surgery has also been reported in literature [12]. However, the use, or no use, of mitomycin C, was not reported in the database. It is well known that mitomycin C modifies the formation of scar tissue, so the lack of reporting use of mitomycin C in the database is a weakness that makes interpretation, of the reported rate of haze, difficult. Elevation of intraocular pressure may be a problem after the use of Phakic IOL AC technique. In our study, this was reported to occur in 2.1% of cases. This is in the lower range of the 2 to 18% that has been reported in literature [13].

The follow-up data should be measured and entered in the system within 1 to 6 months after surgery. This means that refractive outcomes more than 6 months after surgery cannot be captured in the database. The follow-up time decided for the registry was based on pragmatic reasons as longer follow-up means visits later than what is normal routine for many clinics. However, it has been reported in some studies that a long-term follow-up may show refractive changes both after LASIK and PRK.

The data reported in this study show a good refractive outcome for all surgical techniques reported to the database. However, the best-corrected visual outcome could be a concern because refractive surgery is usually performed on healthy eyes with a good preoperative visual acuity. In this study, the surgical techniques used for high myopia resulted in improved best-corrected visual acuity for about a quarter of all operated eyes. For Phakic IOL AC and RLE, the percentage of eyes that gained one line or more after surgery was 23.4% and 25.6%, respectively. Most eyes with a loss of one line or more after surgery were reported for treating hyperopia with Epi-LASIK (37.1%).

The database was meant to offer an opportunity for comparison and bench-marking to the benefit of participating surgeons, clinics, and their patients. The low inflow of data indicates that European refractive surgeons have not detected or appreciated this opportunity. This is in contrast to a number of other ophthalmic databases, which have generated much more interest from ophthalmologists. The quality registry databases for cataract [14,15], corneal transplants [16,17], retinopathy of prematurity [18] and age-related macular degeneration [19] have all been successful. Why is a quality registry for refractive surgery utilized to a lesser extent than other ophthalmic quality registries? One possible reason is lack of awareness of the existence of such a registry and its potential effect on the quality of refractive surgery. However, Dutch refractive surgeons have started to report their surgeries to a national registry (personal communication: Ype Henry). Swedish refractive surgeons will start to report refractive surgeries in 2015 to the EUREQUO (personal communication: Bo Andersén). Another possible reason is that in many cases, even though the surgeon performs the surgery, the referring optometrist/primary care ophthalmologist carries out postoperative follow-up. It is disappointing that the major high street providers of laser refractive surgery have not, to date, contributed to the database.

One weakness of this study is that all data are self-reported by self-selected surgeons interested in reporting to a clinical database. We do not know whether these clinics are a selection of well-performing clinics or just clinics interested in quality control. If the majority of data come from well-performing clinics there may be a bias towards a very good outcome. However, the selection of surgical technique, indications, and trends over time should not be affected. Other weaknesses include the use of different visual acuity test charts and that we do not have data on the use of mitomycin C for surface laser treatments. A strength of this study is the large amount of data from different clinics and countries reflecting outcomes in real life. We hope that the increasing interest in quality improvement among refractive surgeons will result in more reporting to the database in future. Furthermore, with a larger database, more detailed analyses can be performed. The fields for data collection, included in the web form, are currently being reviewed.

## Conclusion

A European registry for refractive surgery has been established. The purpose of the registry is to improve quality of care and serve as a reference database for benchmarking. The number of reported surgical procedures increases with around 4,000 cases added annually. The registry shows changing trends in reported types of surgical procedure but stable indications and good refractive outcomes. The frequency of surgical complications and visual outcomes varies between the surgical procedures.
